# Magnetic Resonance Access to Transiently Formed Protein Complexes**

**DOI:** 10.1002/open.201402008

**Published:** 2014-06-18

**Authors:** Tomáš Sára, Thomas C Schwarz, Dennis Kurzbach, Christoph H Wunderlich, Christoph Kreutz, Robert Konrat

**Affiliations:** [a] Department of Structural and Computational Biology Max F. Perutz LaboratoriesVienna Biocenter Campus 5, 1030 Vienna (Austria) E-mail: robert.konrat@univie.ac.at; [b] Institute of Organic Chemistry and CMBI, University of InnsbruckInnrain 80/82, 6020 Innsbruck (Austria)

**Keywords:** electron paramagnetic resonance, intrinsically disordered proteins, nuclear magnetic resonance, protein–protein interactions, transient complexes, structure characterization of biomolecules

## Abstract

Protein–protein interactions are of utmost importance to an understanding of biological phenomena since non-covalent and therefore reversible couplings between basic proteins leads to the formation of complex regulatory and adaptive molecular systems. Such systems are capable of maintaining their integrity and respond to external stimuli, processes intimately related to living organisms. These interactions, however, span a wide range of dissociation constants, from sub-nanomolar affinities in tight complexes to high-micromolar or even millimolar affinities in weak, transiently formed protein complexes. Herein, we demonstrate how novel NMR and EPR techniques can be used for the characterization of weak protein–protein (ligand) complexes. Applications to intrinsically disordered proteins and transiently formed protein complexes illustrate the potential of these novel techniques to study hitherto unobserved (and unobservable) higher-order structures of proteins.

## Introduction

Protein–protein interactions are fundamental chemical events in living organisms and thus of prime interest to biochemical research in many fields of life science. Although protein X-ray crystallography has delivered an enormous amount of highly refined structural data of large protein aggregates its applicability is limited to high-affinity and stably structured protein complexes. In the recent past, it has yet become clear that also weak protein–protein interactions leading to transiently formed complexes play key roles in diverse biological processes, such as cell signaling, post-translational modifications, general transport phenomena, cellular trafficking, enzyme catalysis, and transcription as well as translational regulation. Additionally, there is growing awareness of the importance of intrinsically disordered proteins (IDPs) in eukaryotic life. Their central role in protein interaction networks has been established. IDPs efficiently sample a vast and heterogeneous conformational space allowing them to interact with multiple and diverse binding partners. It is therefore a great and rewarding challenge for modern structural biology research to access these proteins in the completeness of their native states and to adequately describe their inherent structural plasticity. In this context it is of interest that sparsely populated protein conformational states (high-energy, excited states) are essential for the functionality of all kinds of proteins—ranging from IDPs to tightly structured enzymes.[1–3] It is thus mandated that the conventional notion “protein structure determines function” needs to be reinterpreted (up to now predominantly ground state structures were considered).

Evidently disordered systems are not amenable to X-ray crystallography, which is by far the most widespread structural biology technique. Instead these systems require alternative technological strategies. Nuclear magnetic resonance (NMR) spectroscopy offers unique possibilities for this quest. Because NMR is performed in solution there are different sample requirements. As such, it can also be applied to molecular systems displaying substantial degrees of conformational flexibility. Over the years, NMR methodologies were developed to investigate protein complexes. NMR chemical shift changes and saturation techniques are used to locate protein interaction sites. Residual dipolar couplings (RDC)[4, 5] together with nuclear-Overhauser-effect (NOE)-based methods[6] and paramagnetic relaxation enhancement (PRE)[7] can be used to evaluate binding interfaces with residue resolution, even in cases of weak affinities and transient intermolecular contacts. It should be noted that NMR not only allows for locating interaction sites and provides structural information about the binding interface, but also allows for the quantification of binding affinities (dissociation constants, *K*_D_). For more details and broader coverage of NMR applications, the reader is referred to excellent reviews published over the years.[6, 8, 9] Here we provide an illustration of the power of NMR spectroscopy for the elucidation of reversible protein interaction events by means of so-called relaxation dispersion (Car–Purcell–Meiboom–Gill, CPMG) experiments. Applications to studies of protein–protein interaction are illustrated with a quantitative analysis of Max homodimerization. It is shown that NMR provides detailed information about this dynamic protein dimerization process. Both kinetic and structural parameters are obtained, which permits the analysis of the only marginally populated protein complex. An example for studies of enzyme catalysis is given through the observation of reversible binding of the cold-shock protein CspA to its cognate cold-box RNA. Here, dynamics in unperturbed equilibrium of both, the protein and RNA components, can be studied via NMR spectroscopy. Again, we anticipate that these NMR measurements will provide unprecedented insight into the molecular details and elementary steps of RNA chaperone function. Finally, we present a detailed description of how NMR and electron paramagnetic resonance (EPR) spectroscopy can be used in a complementary fashion to investigate complex formation of IDPs. Most importantly, this novel approach revealed that upon ligand binding the model IDP Osteopontin largely remains disordered although its conformational ensemble is updated. Ligand binding proceeds through conformational selection of a preformed conformational substate that is characterized by an unexpected cooperative stabilization as common for stably folded globular proteins. The observed structural and dynamical compensations upon binding reveal how thermodynamically unfavorable entropic penalties are compensated through partial unfolding of peptide segments remote to the primary binding site.

## Results and Discussion

### Access to sparsely populated conformational states by relaxation dispersion NMR experiments

**Figure 1:**
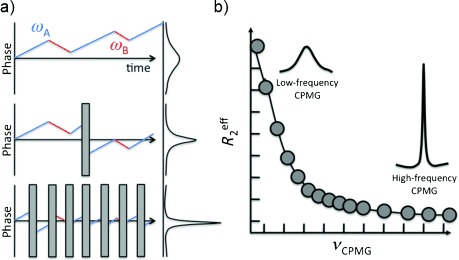
a) Phase of two spins with resonance frequencies *ω*_A_ (—) and *ω*_B_ (—) in dependence of pulse sequence evolution time and pulse interlay in a CPMG experiment. Corresponding detectable NMR signals on the right. With increasing *υ*_CPMG_, the signal becomes sharper. b) Theoretically calculated dependence of *R*_2,eff_ on *υ*_CPMG_.

Biomolecules are characterized by rugged energy surfaces resulting from attractive and repulsive forces of similar strength. The accessible conformational space governs thermodynamics and kinetics of conformational transitions between different substates. In the past, it has become increasingly evident that sparsely populated, high-energy conformational states of a protein can be essential for protein function.[3, 10] A prominent example is the frequently observed conformational selection mechanism for substrate recognition.[11, 12] In this context a substrate is assumed to interact with a (sparsely) populated high-energy (excited) conformation of a protein and to modulate the systems conformational ensemble by stabilizing this particular high-energy state. However, excited conformational states are difficult to access by means of conventional (direct detection) NMR, since fast conformational sampling leads to averaging of resonances. Averaging “hides” a sparsely populated state behind predominating low-energy states. Consider a conformational transition process in which a highly populated (low-energy) ground state exchanges with a lowly populated (high-energy) excited state. The resulting NMR signal is a population weighted average of the individual signals stemming from the two states and dominated by the abundant ground-state signal. Detailed information about the sparsely populated excited state can nevertheless be obtained by the so-called Car–Purcell–Meiboom–Gill (CPMG) relaxation dispersion experiment.[13, 14] This experiment takes advantage of the fact that the conformational transitions between a highly (A) and a lowly (B) populated species affects the transverse relaxation times and thus line widths and intensities of the ground states’ (A) NMR signals. Suppose a 90° pulse that flips the magnetization of an amide nitrogen of a particular amino acid from the quantification axes to the transverse plane. The spin will immediately start to precess and concomitantly dephase during a given time interval *τ*. Conformational transitions between ground and excited state lead to alterations of the precession frequencies (Figure 1, top). Application of a 180° inversion pulse after the time interval *τ* leads to partial refocusing of the precessing magnetization and hence increased signal intensities after the interval 2*τ* (Figure 1, middle). Increasing the number of 180° inversion pulses improves the refocusing efficiency in the CPMG experiment and results in increased signal intensities (Figure 1, bottom). Depending on the overall exchange rate between the two states A and B, *k*=*k*_A_+*k*_B_, (with *k*_A_ and *k*_B_ being the forward and backward rates) the number of refocusing pulses will alter the observed signal intensity. If the exchange rate *k* is slower than 1/*τ*, the precessing magnetization will be completely refocused and signal intensity is unaffected by the exchange process and hence maximized. Yet, if *k* is in the range of or larger than 1/*τ* the observed signal intensity will be modified and depend on the frequency (*υ*_CPMG_) of 180° inversion (refocusing) pulses. In other words, the effective (detected) transverse relaxation rate, *R*_2,eff_ will be affected by the exchange process and the time interval *τ* (or the number of 180° inversion pulses, see Figure 1). Increasing the frequency of 180° inversion pulses, where *υ*_CPMG_=1/(4*τ*), in the CPMG train (Figure 1) will lead to progressive quenching of the exchange process. This dependence is exploited by CPMG pulse sequences in which pulse trains with repeating *τ*–180°_*x*_–τ blocks are applied to refocus the magnetization. Sampling different values of *υ*_CPMG_ yields a dependence of *R*_2,eff_ versus *υ*_CPMG_. *R*_2,eff_(*υ*_CPMG_) will decrease with increasing *υ*_CPMG_ values (Figure 1). In the case of complete quenching of the exchange process, however, one observes a flat profile with *R*_2,eff_=*R*_2,0_ (*R*_2,0_ is the exchange-free transverse relaxation rate). Fitting *R*_2,eff_(*υ*_CPMG_) as a function of *υ*_CPMG_ allows to determine the exchange rate *k* between the two states. Furthermore, the chemical shift difference between A and B and the populations of these states can be extracted through measurements at different magnetic field strengths.[15] Depending on the time scales, different approaches have to be taken into account for quantitative analysis of fast, slow and intermediate exchange regimes.[16]

The CPMG experiment can also be explained from a kinetic point of view by taking into account the deleterious effects of randomly changing precession frequencies on echo formation. The observed NMR signal, that is, a spin echo, will become weaker the more the precision frequencies of the individual spins in an ensemble differ from each other. The time needed for the spins to be randomly distributed in the transverse plane after RF excitation is inversely proportional to the spectrum of precision frequencies (line width). Conformational exchange modulation of precision frequencies due to changes in the local environment of some spins leads to additional line broadening and concomitant signal attenuation. Taking a look at the CPMG pulse train in Figure 1 one can see that this effect will only be effective if the time between two pulses is longer than the spin needs (on average) to change its precession frequency due to conformational changes in the system. In other words, the frequency of refocusing pulses (*υ*_CPMG_) must be shorter than the exchange rate *k* in order to observe enhanced signal attenuation. Thus, by varying *υ*_CPMG_ and observing the consequent effect on the signal intensity one can yield information about *k* and the details of conformational exchange processes (frequency difference between individual states).

In the realm of protein NMR, one frequently observes the relaxation dispersion of backbone ^15^N–^1^H amide resonances. Implementation of the CPMG train into a two dimensional data acquisition yields residue-dependent *R*_2,eff_ values. In this case, a residues exchange frequency might be affected by local conformational interconversion processes, for example, flapping of aromatic side chains,[17] and by global processes like large scale conformational adaptations due to, for example, ligand binding. In order to separate global from local influences, one can choose to fit the dispersion profiles in a global or residue-specific manner. Numerous applications have demonstrated the wide applicability of this approach and provided valuable insight into the atomic details of relevant dynamic processes in biology.[18] Here we illustrate the technique with applications to a transiently formed chaperone–RNA complex and the reversible formation of a homodimeric coiled-coil protein complex.

**Figure 2:**
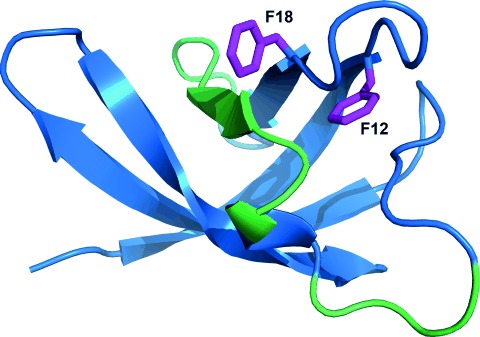
Structural model of CspA with surface hydrophobic residues shown in magenta; the primary RNA binding site is represented in green (residues 26–41). Structure PDB ID: 2L15.[19]

As a case study of lowly populated state of ligand-bound protein, we present here the interaction of the *Escherichia coli* cold-shock protein CspA (the 3D structure of the apo-state of CspA is given in Figure 2) with a fragment of its mRNA. The CspA is a 70 amino acid 7.4 kDa protein naturally occurring in cold-shocked bacteria. It functions as an RNA chaperone by interacting with the 5’-untranslated region (UTR) of its own mRNA (called the cold-box RNA, CB RNA);[20] overproduction of the CspA mRNA causes derepression of the cold-shock genes expression. The CspA structure comprises a β-barrel fold. The RNA binding surface is primarily constituted of solvent-exposed aromatic residues, mostly phenylalanines.[20, 21] However, the RNA-bound state of the protein is difficult to assess by means of directly detected NMR, since only a minor fraction of the present RNA molecules interact with CspA at any given time, and the time frame of the interaction is very short. Saturating the population of the bound state is unfeasible due to the progressive signal loss with increasing RNA concentration as a consequence of significant line broadening, especially in the binding sites.[21] Most other techniques for measuring protein–ligand interaction are not applicable since the population of the bound-state protein is usually below their limit of detection.

**Figure 3:**
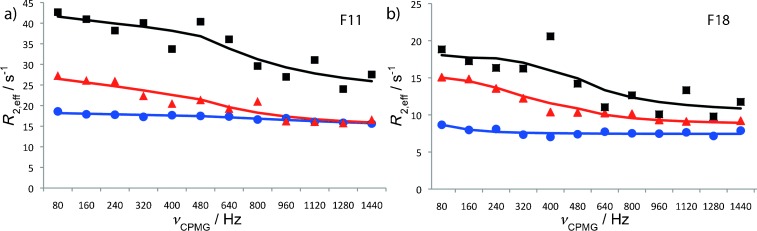
CPMG relaxation dispersion profiles for CspA residues a) F12 and b) F18. Relaxation profiles of the CspA in the presence of 10 % ACB (▲ and▪) and in the absence of RNA (•). Black squares (▪) show the relaxation profile at 800 MHz NMR field frequency and red triangles (▲) show the profile measured at the frequency of 500 MHz; solid lines indicate “global” data fits.

Thus, the CPMG relaxation dispersion (RD) experiment is a promising technique to supply information about the sparsely populated RNA-bound state of CspA. Here, we provide ^15^N-CPMG data of residues F12 and F18 (Figure 3) to illustrate how this technique can be used to analyze the transiently formed CspA–RNA complex. The employed RNA interaction partner is the so-called anti-cold-box (ACB) RNA, which is partially complementary to the cold-box RNA.

For CspA residues F12 and F18, ^15^N-dispersion relaxation profiles were measured in the absence and in the presence of ACB RNA (Figure 3). In the absence of RNA, no exchange process can be detected as reflected in the flat dispersion relaxation profiles. However, in the presence of 10 % molar ratio of ACB RNA, distinct changes in relaxation profiles were observed. Global fitting based on an intermediate exchange model,[16] yielded 1.6 % of the conformational ensemble to comprise the CspA RNA-bound state; the exchange frequency was determined to be 1.4 kHz. The participation of the phenylalanine residues F12 and F18 (highlighted in Figure 2) adjacent to the primary RNA binding site during the binding event was previously described.[21] It is noteworthy that the relaxation dispersion profiles not necessarily reflect the kinetics of the RNA binding event, but the frequency of the structural rearrangements in the region comprising residues F12 and F18. However, it has been shown that the RNA binding triggers structural adaption of the protein. Hence, it is reasonable to correlate the exchange frequency (1.4 kHz) fitted globally to F12 and F18 with the effective time constant of the RNA binding/release. Referring to the structural model shown in Figure 2, the F12 and F18 residues are positioned sideways to the primary binding site and can therefore establish stacking interactions with the ribose moieties in the bound RNA strand. Other residues not present in either the primary or the secondary binding site do not exhibit changes in *R*_2,eff_ as observed for F12 and F18 (data not shown). Thus, CPMG analysis allows for determination of kinetics of a state as lowly populated as 1.6 %, which would be difficult to assess by other techniques. Further, the method used here enables one to obtain information not only on kinetics and population, but also yields the nitrogen chemical shift differences between the free and the bound state of residues observed by CPMG data that correlate with the magnitude of changes in the chemical environment. Thus, in principle one could extract structural information about the bound state of CspA by means of CPMG through the chemical shift difference to the apo-state if a sufficiently high number of residues yield exchange-affected dispersion profiles. The CspA case study presented here demonstrates the power and versatility of the CPMG method in assessing the lowly populated states important in RNA–protein interactions.

**Figure 4:**
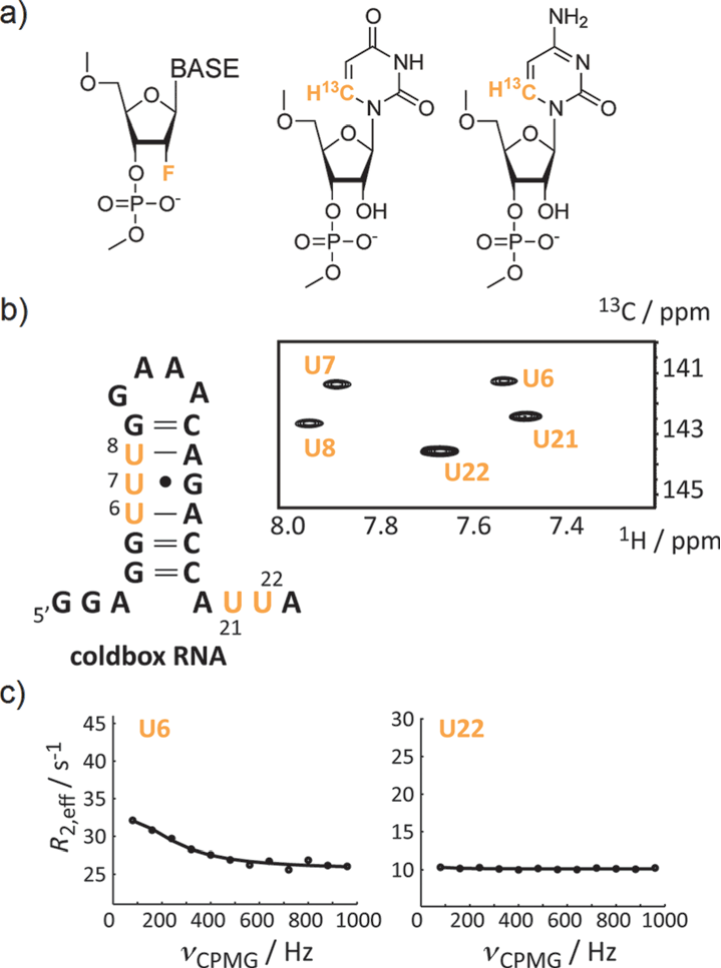
a) Stable isotope labeling patterns that can be introduced via the solid-phase RNA synthesis approach. 2′-Fluorine and 6-^13^C-pyrimidine labels are shown. b) Cold-box RNA with five 6-^13^C-uridine labels and the 1H–^13^C-HSQC spectrum with assignments. c) Selected ^13^C-CPMG relaxation dispersion profiles at 125 MHz carbon larmor frequency of the cold-box RNA. Residue U6 shows a non-flat dispersion profile indicating microsecond to millisecond dynamics, whereas U22 displays no dynamics on that timescale.

To complement the results obtained from CspA-derived CPMG measurements, we also focused on the investigation of micro- to millisecond dynamic processes of the CB and ACB RNAs, which interact with CspA. In contrast to protein NMR spectroscopy, where the backbone amide nitrogen proton spin pairs represent suitable reporter nuclei for CPMG RD experiments, for nucleic acids, ^13^C-CMPG RD experiments are the method of choice.[22] This is due to the fast exchange rates of the imino protons, that is, nitrogen-bound protons (H3 in uridine and H1 in guanosine) directly involved in base pairing, with bulk water leading to significant line-broadening effects or even to a total collapse of the imino proton signal within the dominant water resonance. But as in protein NMR spectroscopy, uniform ^13^C/^15^N-labeling patterns impair the application of certain NMR experiments to address dynamic features, like the CPMG RD experiment. A uniform ^13^C/^15^N-labeling pattern introduces one-bond scalar and dipolar ^13^C–^13^C couplings resulting in resolution and sensitivity problems especially for larger RNA comprising more than 30 nucleotides (nt) but also to artifacts in the CPMG RD data sets impairing a reliable analysis.[23] To circumvent several issues arising from the state-of-the-art uniform labeling protocol using ^13^C/^15^N-modified ribonucleotide triphosphates (rNTPs) and T7 RNA polymerase, several approaches were proposed.[24] In one of our laboratories, we have recently put focus on isotope labeling RNAs via the solid-phase synthesis approach. For example, a minimally invasive ^19^F-based isotope labeling protocol can be very efficiently applied to RNA as all four 2′-fluorine modified nucleotide phosphoramidites are commercially available and can be incorporated into the target RNA without modifying the standard protocol (Figure 4). The ^19^F nucleus has favorable NMR spectroscopic features, like 100 % natural abundance, an intrinsic NMR sensitivity almost as high as protons (83 %) and a vast chemical shift range about 100 times larger than protons. It represents a bioorthogonal reporter spin for nucleic acids and was efficiently used in RNA folding and RNA ligand binding NMR assays.[5–27] We currently introduce 2′-fluorine labels into the CB and ACB RNAs to study the interaction of both RNAs with CspA but also to address conformational dynamics via ^19^F-CPMG relaxation dispersion experiments (data not shown). We also focused on the chemical synthesis of site-specifically ^13^C-modified pyrimidine phosphoramidites (Figure 4).[28] This labeling protocol results in functionally unperturbed RNAs at the costs of the labor-intensive production of the 6-^13^C-uridine and -cytidine phosphoramidites. For example, all uridines in the aforementioned stem-loop motif (CB RNA, Figure 4**)** residing in the 5′-UTR of the *E. coli* cspA messenger RNA (mRNA) were replaced by the 6-^13^C-modified counterparts. We then used ^13^C-CPMG relaxation dispersion experiments to address the micro- to millisecond dynamics of this very RNA. Preliminary results are shown (Figure 4) and hint towards a dynamic hotspot of the cold-box RNA in the vicinity of the G**⋅**U wobble base pair. A quantitative description of this dynamic phenomenon and the possible biological relevance for the interaction with CspA are currently investigated in our research groups.

**Figure 5:**
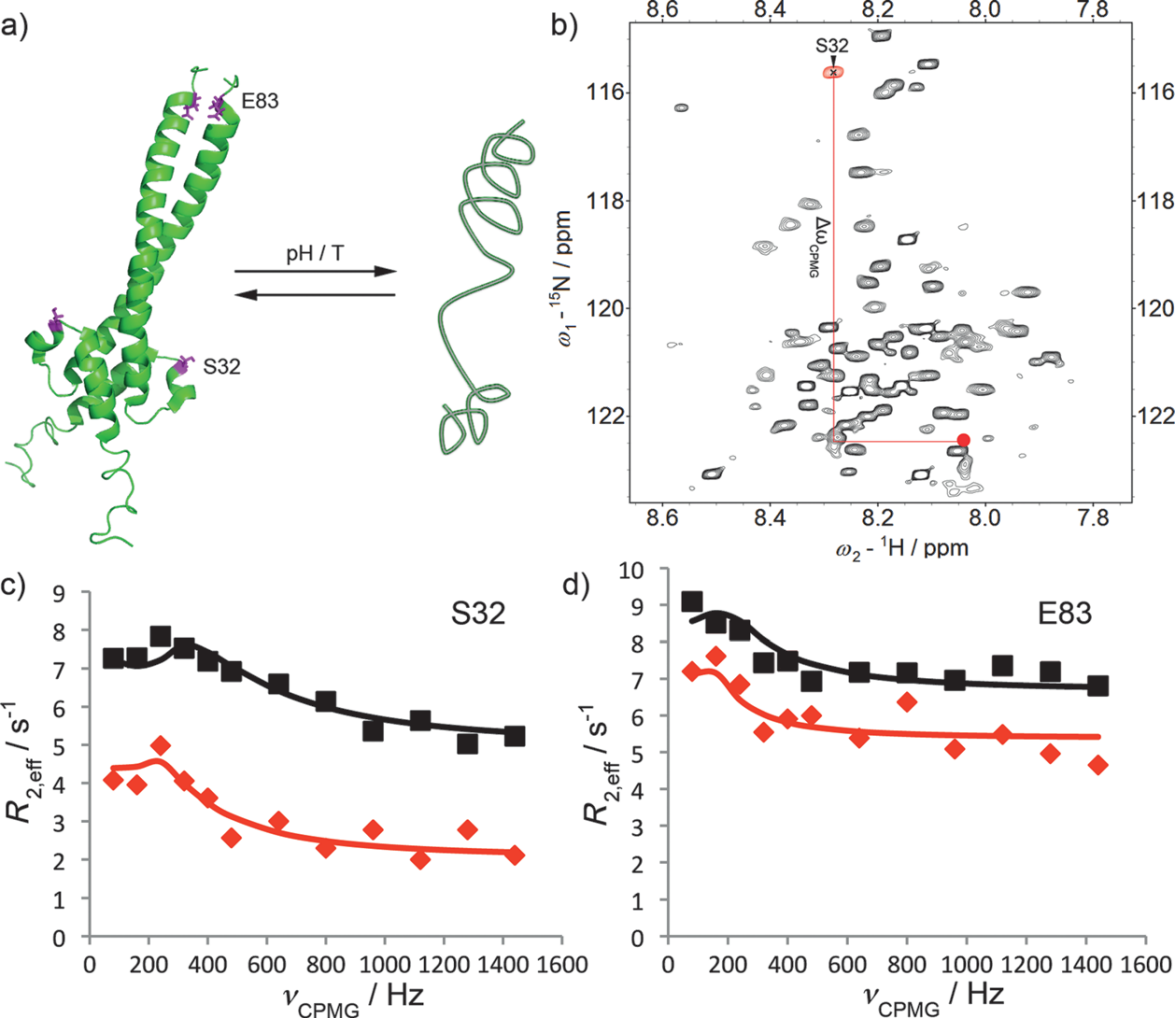
a) Sketch of the dimerization equilibrium of MAX. b) ^15^N–^1^H HSQC of the monomeric state of MAX. The red dot indicates ^15^N and ^1^H frequencies for residue S32 in the homodimeric coiled-coil state (taken from ref. [29]). c,d) CPMG relaxation dispersion profiles at 500 (red) and 800 (black) MHz. c) Residue Ser32 d) Residue Glu83. The solid lines represent fits of the data with an intermediate exchange model.

As a third example for the application of CPMG techniques to transient protein interaction studies, we outline an application to the reversible protein dimerization of the Myc-associated factor X (MAX).[29] MAX readily forms heterodimers with its authentic binding partner Myc yielding an active transcription factor complex. Deregulation of this tightly controlled protein complex formation can lead to severe disease phenotypes.[29] Under neutral pH conditions and room temperature, MAX natively populates predominantly a dimeric, coiled-coil α-helical motif (see Figure 5).[30] Changing solution conditions such as pH and temperature, however, allows for controlled manipulation of the monomer–dimer equilibrium.[30] In Figure 5, a ^15^N–^1^H HSQC of MAX is shown under conditions that favor the monomeric form (pH 5.5, 35 °C).[30] The strong spectral overlap indicates that the prevailing monomeric form of MAX is to a large degree conformationally disordered. In Figure 5 we show CPMG profiles of two residues, Ser32 and Glu83, of MAX under these conditions at 500 and 800 MHz NMR static field strength. The observed exchange process corresponds to interconversion of monomer and dimer MAX states.[30] Fitting the data globally yields a population of the MAX dimer of 0.75 % with an exchange rate of 0.4 KHz.[16] This is in excellent agreement with data obtained through a thermodynamic analysis of the MAX monomer–dimer equilibrium.[30]

The reliability of the CPMG results can be further tested by comparison of the chemical shifts extracted from the CPMG data with literature data for the dimeric state of MAX. From earlier publications, for example, the *ω*_N_ of the bound (dimeric) state of MAX is known, while the chemical shift of the monomeric form can be directly observed in ^15^N–^1^H HSQC spectra (since the monomeric form of MAX is the dominating species under these conditions). The differences between monomeric and dimeric chemical shifts should coincide with individual *δω*’s obtained from a fit of CPMG data. As indicated in Figure 5, for Ser32 excellent agreement between fitted and experimental chemical shift differences were found (*δω*_fit_=8.0 ppm and *ω*_N,Dimer_−*ω*_N,Monomer_=7.8 ppm).[29] Because the chemical shift change depends on molecular rearrangements (e.g., through changes in conformation, local hydration, hydrogen bonds etc.), unique information can be obtained about the atomic details of sparsely populated protein states even under conditions where other experimental techniques fail as a consequence of the low population.

### Assessing IDP substates by means of combined EPR and NMR

The hallmark of intrinsically disordered proteins (IDPs) is their vast and heterogeneous conformational ensemble comprising both extended conformations and simultaneously compact reasonably stable structures displaying significant resistance against denaturing agents (like urea).[31] The rugged energy surface typically found for IDPs allows for conformational transitions between different substates and endows IDPs to transiently interact with a multitude of binding partners via, for example, conformation selection type processes. Despite their enormous biological relevance, the structural and dynamical characterization of this important protein family is still far from routine. Most importantly, the existence of sparsely populated substates in the conformational ensemble requires highly sensitive experimental techniques to probe their structural features and encounters with protein binding partners. It was recently demonstrated that electron paramagnetic resonance (EPR) spectroscopy is a very powerful experimental tool to access sparsely populated states and transient protein interactions.[32–35] Furthermore, EPR spectroscopy is a promising candidate to supplement NMR data in terms of time and length scales. Some pilot studies of EPR on IDPs have already been published,[32–35] yet the strength of EPR, that is, the detection of through-space dipolar interaction of electron spins by double electron-electron resonance (DEER) spectroscopy,[36–39] has turned out to be difficult to apply to IDPs. The commonly applied analysis techniques for DEER raw data fail in the case of IDP conformational ensembles.[40] Therefore, we have amended the repertoire of analysis methods for DEER data with an approach that is suitable to interpret data obtained for IDPs comprising large and heterogeneous conformational ensembles. In the following section, we will briefly introduce the standard DEER methodology and explain our expansion of this method to IDPs. Our investigations based on this novel approach are accompanied by complementary NMR experiments. Generally speaking, we show how NMR and EPR data supplement each other and provide a comprehensive picture of IDPs’ structural dynamics.

In order to perform DEER, one has to modify a protein with two labels that both carry an unpaired electron, hence, the term spin label.[41] The free electrons of the two labels will experience a through space dipolar coupling. However, since at room temperature dipolar couplings between two labels in solution average to zero, the sample has to be freeze-quenched. As such, one measures the intramolecular dipolar couplings between every pair of spin labels “locked in” at the conformation at the glass transition temperature of a sample (after correcting for intermolecular background contributions). In order to prolong relaxation times, DEER is, yet, measured at even lower temperatures, typically between 20 and 50 K. The dipolar couplings manifest themselves as modulation of DEER time domain data, that is, the signal intensity as a function of the pulse sequence evolution time.[31, 38, 42] For a single, fixed distance, one would get a single cosine modulation of the time trace. Yet, as the conformational ensemble grows, the number of populated substates increases, and each substate contributes to the DEER time trace. The frequency of the cosine modulation is dependent on the interspin distance. For the common DEER analysis methods, the individual contributions of the different substates are given primarily by their weighted populations (note that average interspin distances can become of importance in large disordered systems, too).[31] With increasing number of populated conformations and corresponding interspin distances the total, observable modulations will become increasingly blurred and will ultimately converge to an exponential decay. This circumstance gives rise to particular problems in separating intra- and intermolecular contributions to the DEER signal for broad distributions of distances. In the case of doubly labeled proteins, one typically wants to eliminate intermolecular contributions. For intrinsically disordered systems, this can only be achieved by measuring DEER references on singly spin-labeled proteins, which yields the pure intermolecular contributions.[41]

**Figure 6:**
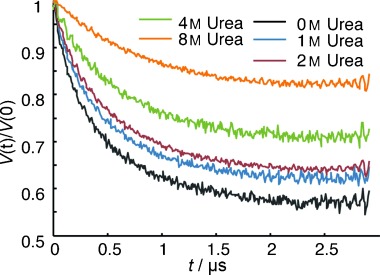
DEER time traces of a double mutant comprising the central OPN segment from residue C108 to C188 at different urea concentrations.

In case of folded proteins, after removal of background contributions, the spin label distance distribution *P*(*R*) can be obtained by well-established procedures such as the Thikonov regularization.[41, 43] Yet, in the case of IDPs the broad and inhomogeneous distance distributions leads to less significant time trace modulations and poor signal-to-noise ratios that impairs the extraction of feasible distance distributions (e.g., like the traces shown in Figure 6).[40] Thus, we proposed an alternative approach based on a so-called effective modulation depth, *Δ*_eff_, for data interpretation of IDPs. Details of this novel approach were explained elsewhere.[31] In short, *Δ*_eff_ denotes the signal decay of the time trace, *V*(*t*), at a given point of time, *t*_eff_. *Δ*_eff_ can be defined as: *Δ*_eff_=1−*V*(*t*_eff_)/*V*(*t*=0). For large IDPs, we suggest to simply choose the longest experimentally achievable DEER evolution time for *t*_eff_. *Δ*_eff_ is an approximate measure of average interspin distance for broad *P*(*R*)s. Yet, the reader should be aware that *Δ*_eff_ does not linearly depend on the population-weighted average distance in the measured ensemble but is a complex function of several spectroscopic parameters.[31] To a first approximation, however, *Δ*_eff_ decreases with increasing interspin distance *R* for broad distance distributions. With *Δ*_eff_ as tool in hand, we probed structural preferences of Osteopontin (OPN), a cytokine involved in metastasis of several kinds of cancer.[44] Typical DEER data obtained on an IDP are shown in Figure 6.

**Figure 7:**
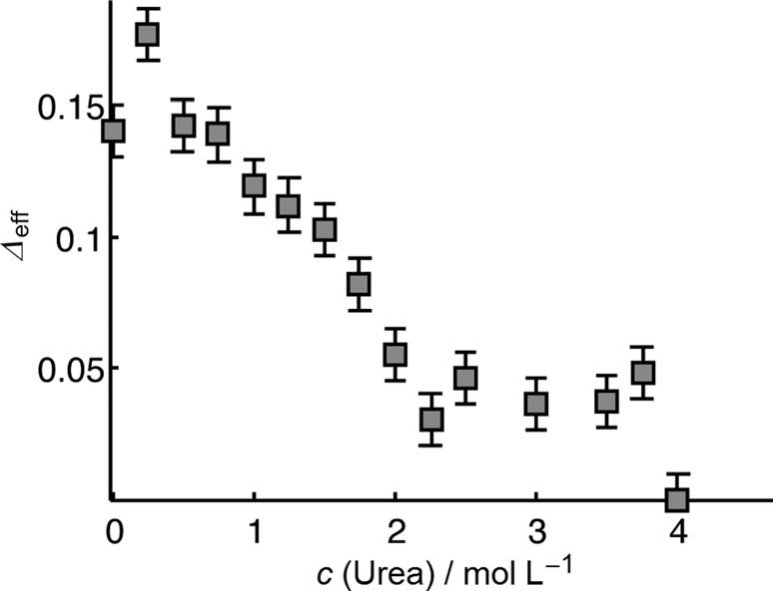
*Δ*_eff_ for an OPN mutant (C54–C247) comprising nearly the whole protein as a function of urea concentration. Error bars stem from signal noise.

Conformational stabilities, understood as resistance to urea unfolding, of several individual structural segments of OPN were investigated by recording DEER time traces for different spin labeled double mutants in dependence of increasing urea concentration. As such, a decrease of *Δ*_eff_ with increasing urea concentration is representative for unfolding and expansion of a doubly spin-labeled protein of interest. In Figure 6 exemplarily experimental *Δ*_eff_ values are shown as a function of urea concentration for a selected double mutant comprising a central segment of the cytokine OPN. The time traces appear as exponential decays because of the aforementioned convergence of cosine functions (one for each conformation in the OPN ensemble). With increasing urea concentration *Δ*_eff_ decreases indicating an expansion of the IDP. As published earlier,[31] the C-terminal part of OPN exhibits an exponential decay of *Δ*_eff_ with increasing urea concentration. This gives rise to a steep slope of the *Δ*_eff_ function and can be regarded as a denaturation profile of an unstably folded protein segments of potentially random-coil-like character. Already for low urea concentrations such segments show significant conformational expansion (i.e., a decrease in *Δ*_eff_) in accordance with the idea of very low stability of transient or residual structural elements in IDPs. For other mutants, one might observe an approximately linear decrease of *Δ*_eff_ with urea concentration, indicating that the OPN segment framed by these mutants is on average conformationally more stable than the C-terminal segment. Nevertheless, it is still largely unstructured, random coil- or (pre)molten globule-like. Strikingly, however, for a mutant of OPN with two terminal labels, we observe a sigmoidal development of the *Δ*_eff_-derived denaturation profiles with urea concentration (see Figure 7). Sigmoidality is a hallmark for cooperative folding of protein conformations and unexpected for an IDP.[45]

A sigmoidal denaturation profile is indicative for stably and cooperatively folded tertiary structures of OPN, since for low urea concentrations of up to 0.75 m the whole protein does not expand significantly (as seen in nearly constant *Δ*_eff_ values). This observation of a cooperatively folded conformation is surprising since distance distributions between two labeled residues of OPN are generally quite broad, as deducible from prior studies concerning OPNs conformational space[11] and as reflected in the non-modulated DEER time traces (Figure 6). This interesting finding can, however, be understood by concluding that the structural ensemble of OPN contains both cooperatively folded and unfolded conformations and that both contribute to the DEER signals.[31] This deduction is possible here only because DEER EPR on freeze-quenched solutions elucidates the whole set of co-existing conformations; ensemble averaged data here would not allow for discerning between partial structuring and sampling of compact conformations. Most importantly, the existence of structural cooperative transitions from folded to unfolded states and vice versa as monitored by EPR in combination with NMR in IDPs calls for a novel conceptual view of IDPs that goes beyond the traditional binary scheme of order versus disorder. The subtleties of heterogeneous conformational sampling in IDPs and their putative relevance for biological functions have to be adequately addressed.

**Figure 8:**
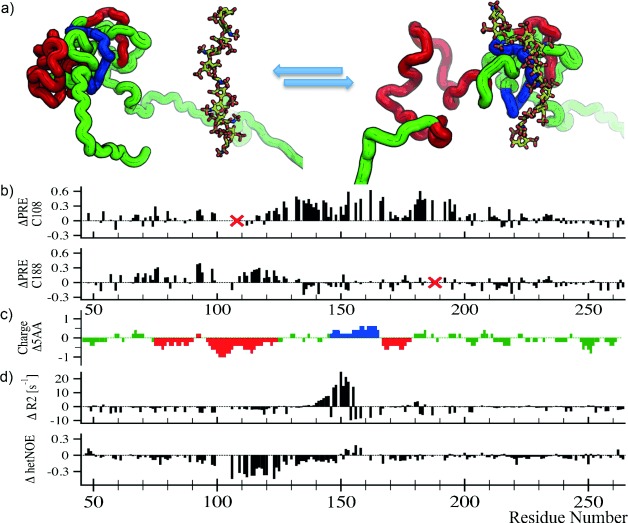
a) Schematic representation of the OPN—heparin interaction. b) Differential PRE values for the two central mutants (PRE of OPN and heparin; PRE of OPN). The positions of spin labels are indicated (red crosses). c) Approximate residue charge of OPN. The color code is identical to the schematic representation of the binding process in Figure 8. d) Differential transverse relaxation and heteronuclear NOEs (OPN and heparin; OPN).

The applicability of this novel EPR-NMR approach to the observation of transient complex formation of IDPs was also recently demonstrated. Not only can the *Δ*_eff_ approach be utilized to elucidate structural preferences of IDPs complementary to NMR data, but also ligand interaction can be observed in high detail. We analyzed the interaction of OPN with heparin,[11] a highly sulfated glycosaminoglycan widely used as anticoagulant (see Figure 8).[10] In a biological context, heparin binding to OPN is of interest since it models the OPN–heparan sulfate interaction, which constitutes a crucial cofactor in OPN–CD44 receptor association, a process involved in cell signaling and adhesion.[46] Additionally, interactions between IDPs and biological polyelectrolytes are quite common,[3] and our results might well be applicable to other systems.

Upon binding to heparin, OPN largely remains disordered although its structural ensemble is updated. For several doubly spin-labeled mutants of OPN, heparin binding leads to a clear decrease in *Δ*_eff_ indicating longer distances between the labeling sites of the six double mutants and an expansion of the protein upon heparin binding. This is shown in a previous paper.[10] This information from EPR agrees well with information concerning the binding event gained from NMR-based paramagnetic relaxation enhancements (PREs).[10] When interpreting PREs of IDPs, one should be aware that due to the rapid conformational sampling of IDPs one observes ensemble averaged PRE data.[47] Hence, all conclusions drawn from these refer to “average” conformations and differential values (bound minus apo-state PREs), ΔPRE>0 indicate “on average” increasing distance in the protein complex between labeling site and a residue upon binding, ΔPRE<0 the opposite. As can be observed in Figure 8, OPN displays differential changes of long-range backbone interactions as heparin binds. The central spin label attached to C108 experiences a displacement from the core region around residues 130–190, whereas the spin label C188 is separated from the region comprising residues 90 and 120. Information on the binding process and its impact on OPNs dynamic behavior can be obtained when comparing the ^15^N NMR relaxation rates and heteronuclear ^15^N–^1^H NOEs (hetNOE) of bound and free forms. NMR relaxation reports on motions ranging from picosecond to low-nanosecond timescales. The three measurable NMR relaxation parameters (*R*_1_,*R*_2_ and hetNOE; Figure 8) have different dependencies on the time scales of motions. While the ^15^N transverse relaxation rate *R*_2_ reports on nanosecond motions, the heteronuclear ^15^N–^1^H NOE depends on the efficiency of magnetization transfer from ^15^N to ^1^H in the protein backbone, and which is mostly influenced by very fast (picosecond time scale) dynamics of the N−H bond. Figure 8 shows that upon heparin binding to OPN, very fast motions (probed by ^15^N–^1^H hetNOE) increase in the region located around residue 120. The central region around residue 150, however, shows increased ^15^N transverse relaxation rates *R*_2_ due to decreased conformational flexibility in the binding cleft. Closer inspection of the charge distribution in OPNs primary sequence reveals that the differential mobility changes upon heparin binding are linked to the electrostatic pattern found in OPN (increased mobility in negatively charged regions and decreased mobility/rigidification in positively charged patches).

Through combination of NMR and EPR a clear picture arises of the OPN–heparin binding event. As the highly negatively charged heparin contacts OPNs positively charged core region, the structural and dynamic properties of the OPN ensemble get drastically altered. Along with an increase in dynamics along most of the proteins backbone, an average increase in distances is observed. This unfolding-upon-binding event is depicted in Figure 8. The interaction of heparin (sticks) with OPNs positive core (blue) leads to a displacement of the N-terminal negatively charged region (red) either by direct repulsion through heparin or by loss of contacts to the positive region, explaining both the average increase in distances and the dynamic changes.

From isothermal titration calorimetry (ITC) measurements, Δ*H* and Δ*S* values of −16.3 kCal mol^−1^ and −35 cal mol K^−1^, respectively, can be obtained for the heparin binding event of OPN assuming an average molecular weight of 17.5 kDa for heparin. These quite large Δ*H* and Δ*S* values nearly cancel each other at 293 K in the Gibbs energy (Δ*G*=Δ*H*−*T*Δ*S*). Thus, although there is local rigidification in the heparin binding cleft (region around residue 155) the resulting conformational entropy penalty is reduced by a compensatory increase in conformational flexibility of the negatively charged region 90–120. The local (or segmental) unfolding and expansion of the OPN core segment thereby significantly contributes to the overall thermodynamic equilibrium balanced between counteracting contributions like solvation enthalpy, rotational and translational degrees of freedom and conformational entropy.[48, 49]

## Conclusions

Three examples for weak and reversible protein interactions are presented in combination with magnetic resonance based experimental techniques to access these interactions. Relaxation dispersion (Car–Purcell–Meiboom–Gill, CPMG) experiments allow for determination of sparsely populated states in CspA–RNA complexation and MAX–MAX homodimerization. Since both kinetic data about complex formation (association and dissociation rates) as well as structural details of lowly populated (high-energy) conformational states are accessible by this technique, unique information about protein interactions becomes amenable. Further, the combination of paramagnetic relaxation enhancement (PRE) NMR and double electron-electron resonance (DEER) electron paramagnetic resonance (EPR) gives rise to the unprecedented and unexpected observation of cooperatively folded conformational states contained in the conformational ensemble of Osteopontin (OPN) and its changes in the weak OPN–heparin interaction. Our findings suggest that more elaborate conceptual approaches are required for an adequate description of intrinsically disordered proteins and their conformational ensembles.

Proteins are characterized by significant structural plasticity and can undergo large structural rearrangements of the time-averaged conformational ensemble. It is therefore evident that classical structural biology approaches are only starting points for a comprehensive analysis of protein function. For intrinsically disordered proteins (IDPs) flat energy landscapes allow for rapid exchange between different conformational isomers (substates). These often only sparsely populated conformational states, yet, lead to the formation of protein complexes that are essential for biological function. Detailed knowledge of the transiently formed intermediates along the reaction trajectory will be highly valuable. Further developments of new techniques but also the amendment of existing ones (as illustrated with the novel analysis technique of DEER data) will be necessary to provide a more complete picture of how proteins form biologically active molecular aggregates and perform the myriad of essential tasks and how this information can be exploited to manipulate their activities based on knowledge of the underlying chemical mechanisms.
